# Generating Operative Workflows for Vestibular Schwannoma Resection: A Two-Stage Delphi's Consensus in Collaboration with the British Skull Base Society. Part 2: The Translabyrinthine Approach

**DOI:** 10.1055/s-0042-1755578

**Published:** 2022-10-10

**Authors:** Hugo Layard Horsfall, Danyal Z. Khan, Justin Collins, Stephen Cooke, Simon R. Freeman, Nihal Gurusinghe, Susie Hampton, Carl Hardwidge, Richard Irving, Neil Kitchen, Andrew King, Sherif Khalil, Chan H. Koh, Colin Leonard, Hani J. Marcus, William Muirhead, Rupert Obholzer, Omar Pathmanaban, Iain J. A. Robertson, Jonathan Shapey, Danail Stoyanov, Mario Teo, James R. Tysome, Patrick Grover, Shakeel R. Saeed

**Affiliations:** 1 Victor Horsley Department of Neurosurgery, National Hospital for Neurology and Neurosurgery, London, United Kingdom; 2 Wellcome/EPSRC Centre for Interventional and Surgical Sciences, University College London, London, United Kingdom; 3 Department of Urooncology, University College London Hospitals National Health Service Foundation Trust, London, United Kingdom; 4 Department of Neurosurgery, Belfast Health and Social Care Trust, Belfast, United Kingdom; 5 Department of Otolaryngology, Manchester Centre for Clinical Neurosciences, Salford Royal Hospital, Salford, United Kingdom; 6 Department of Neurosurgery, Lancashire Teaching Hospital, Preston, United Kingdom; 7 Department of Ear, Nose and Throat, Belfast Health and Social Care Trust, Belfast, United Kingdom; 8 Department of Neurosurgery, University Hospital Sussex, Brighton, United Kingdom; 9 Ear, Nose and Throat, Queen Elizabeth Hospital, Birmingham, United Kingdom; 10 Geoffrey Jefferson Brain Research Centre, Manchester Academic Health Science Centre, Manchester, United Kingdom; 11 Northern Care Alliance National Health Service Group, University of Manchester, Manchester, United Kingdom; 12 The Royal National Throat, Nose and Ear Hospital, London, United Kingdom; 13 Department of Neurosurgery, Manchester Centre for Clinical Neurosciences, Salford Royal Hospital, Salford, United Kingdom; 14 Department of Neurosurgery, Nottingham University Hospitals, Nottingham, United Kingdom; 15 Department of Neurosurgery, Kings College Hospital, London, United Kingdom; 16 Bristol Institute of Clinical Neuroscience, Southmead Hospital, Bristol, United Kingdom; 17 Department of Ear, Nose and Throat, Cambridge University Hospitals, Cambridge, United Kingdom

**Keywords:** retrosigmoid, translabyrinthine, vestibular schwannoma, skull base surgery, consensus, Delphi

## Abstract

**Objective** An operative workflow systematically compartmentalizes operations into hierarchal components of phases, steps, instrument, technique errors, and event errors. Operative workflow provides a foundation for education, training, and understanding of surgical variation. In this Part 2, we present a codified operative workflow for the translabyrinthine approach to vestibular schwannoma resection.

**Methods** A mixed-method consensus process of literature review, small-group Delphi's consensus, followed by a national Delphi's consensus was performed in collaboration with British Skull Base Society (BSBS). Each Delphi's round was repeated until data saturation and over 90% consensus was reached.

**Results** Seventeen consultant skull base surgeons (nine neurosurgeons and eight ENT [ear, nose, and throat]) with median of 13.9 years of experience (interquartile range: 18.1 years) of independent practice participated. There was a 100% response rate across both the Delphi rounds. The translabyrinthine approach had the following five phases and 57 unique steps: Phase 1, approach and exposure; Phase 2, mastoidectomy; Phase 3, internal auditory canal and dural opening; Phase 4, tumor debulking and excision; and Phase 5, closure.

**Conclusion** We present Part 2 of a national, multicenter, consensus-derived, codified operative workflow for the translabyrinthine approach to vestibular schwannomas. The five phases contain the operative, steps, instruments, technique errors, and event errors. The codified translabyrinthine approach presented in this manuscript can serve as foundational research for future work, such as the application of artificial intelligence to vestibular schwannoma resection and comparative surgical research.

## Introduction

In Part 1 of this series we generated, through expert Delphi's consensus, a codified operative workflow for the retrosigmoid approach to vestibular schwannoma 1 . An operative workflow systematically deconstruct complex procedures into defined tasks and errors. 2 3 The surgical procedure is broken down into phases which contain a series of steps, generating the operative workflow. 3 Existing literature has demonstrated subject experts generating comprehensive and standardized operative workflows. 4 5 6 7 Practical benefits of consensus-driven operative workflows include: (1) workflow analysis; (2) training; (3) creation of high-fidelity simulation models; (4) objective assessment of procedure-specific surgical skills; (5) evaluation of novel technologies or techniques; (6) operating room efficiency improvements. 3 5 8 9

There remains variability between surgeons and centers on how to perform the translabyrinthine approach to resect vestibular schwannoma, including surgeon preference or tumor location and characteristics, all of which can result in different operative outcomes. 10 11 12

In Part 2, we herein present an operative workflow for translabyrinthine approach for vestibular schwannoma, through an expert consensus process in collaboration with the British Skull Base Society (BSBS). This operative workflow aimed to digitize the approaches and provide foundational research in which to build, for example, the application of artificial intelligence to vestibular schwannoma resection.

## Methods

### Overview

The methodology was drawn from previous work from our group and was completed in parallel to the retrosigmoid operative workflow generation 1 6 . This process aimed to generate a comprehensive workflow framework which captured how each approach could reasonably be performed. We did not aim to dictate how an operation should be done. The beginning of the operation was taken as the first incision, adhering to the American College of Surgeon's definition of surgery, “structurally altering the human body by the incision or destruction of tissues.” 13 Therefore, variation relating to position of the patient and incision analysis was not within the scope of this work, although the authors recognize that positioning plays a critical role for any given procedure. The components for workflow analysis and associated definitions are listed in Table 1 . Expert input will be derived through an iterative, mixed-methods consensus process ( Fig. 1 ).

**Fig:**
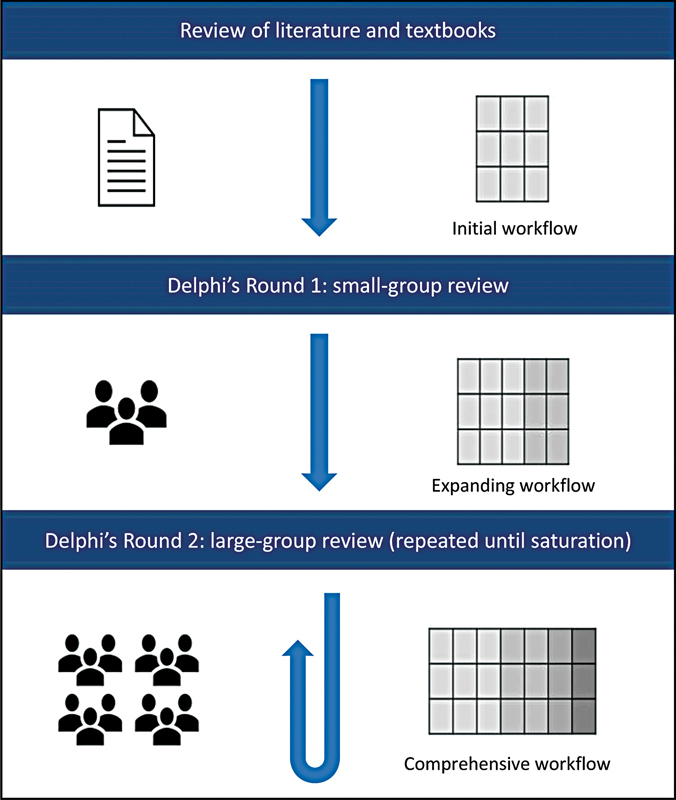
**. 1** Schematic diagram of Delphi's process, highlighting the generation of a surgical workflow through iterative consensus from British Skull Base Society expert members. 1 Adapted from Marcus et al. 7

**Table 1:** Definition of operative workflow terminology per domain

Domain	Definition	Example
Phase	A major event occurring during a surgical procedure, composed of several steps 7	Approach and exposure, encompassing the beginning of surgery until tumor debulking
Step	A sequence of activities used to achieve a surgical objective 24	Seal mastoid air cells
Instrument	A tool or device for performing specific actions (such as cutting, dissecting, grasping, holding, retracting, or suturing) during a surgical step	Bone wax
Technical error	Lapses in operative technique while performing a surgical step 25	Failure to seal mastoid air cells
Adverse event	An intraoperative event which is a result of a technical error and has the potential to lead to a postoperative adverse outcome/complication 25	Cerebrospinal fluid rhinorrhea

### Modified Delphi's Process and Sampling

#### Literature Review

We performed a literature review of Greenberg's Handbook of Neurosurgery, Youmans and Winn Neurological Surgery, and Operative Cranial Neurosurgical Anatomy, and undertook a PubMed and EMBASE search using the keywords “retrosigmoid,” “translabyrinthine,” and “vestibular schwannoma resection” 10 14 15 16 17 18 ( Fig. 1 ).

#### Delphi's Round 1

The initial literature-based operative workflow was reviewed by a group of five consultant skull base surgeons including neurosurgery and ear nose and throat (ENT), based at the National Hospital for Neurology and Neurosurgery, London, United Kingdom. Each consultant surgeon reviewed the operative workflow individually, via computerized document with the definitions of phases, steps, instruments, technical errors, and adverse events as above ( Table 1 ). Each expert was asked a series of questions via e-mail, seeking to assess the completeness and accuracy of the workflow ( Supplementary Material A , available in the online version). 7 Any additional suggestions were reviewed and added to the workflow matrix if in scope and not duplicate. According to the Delphi technique, circulation and iterative revision of the workflow was repeated until data saturation was achieved, that is, all experts were satisfied that the operative workflow was complete and accurate. 7

#### Delphi's Round 2

The refined workflow was circulated nationally with skull base surgeons (neurosurgeons and ENT) who were members of the BSBS, 19 the United Kingdom and Ireland's society primarily focused on skull base pathology. The entirety of the BSBS was invited to participate via e-mail. All contributing authors are specialist lateral skull base surgeons with an independent surgical practice in vestibular schwannoma surgery who are members of the BSBS (either neurosurgery or ENT). Consultant surgeon members from the BSBS were asked to assess the workflow and suggest any amendments to encompass possible variation in practice and technique. Additional suggestions were reviewed and added to the workflow if (1) in scope and (2) not duplicate. 7 Round 2 was completed until all surgeons agreed that the workflow captured the operative practice and that there were no additional suggestions for the workflow from the participant group. Both the retrosigmoid and translabyrinthine approaches were completed in parallel: surgeons within the BSBS were given the opportunity to contribute to either approach depending on their personal clinical practice and expertise. Experience for all authors was calculated from the date they were added to the General Medical Council's Specialist Register, a list of doctors who have completed their postgraduate training and eligible to work as a consultant. 20

### Administration

Invitations to participate in the Delphi process were sent via direct e-mail only. Workflow documents were presented using Microsoft Word (Version 16.4, Microsoft, United States) in both rounds and supported by Google Forms in Round 2 (Google LLC, United States).

### Data Collection and Analysis

Participant demographics collected included surgical specialty and unit. The collected data regarding the surgical workflow were quantitative (whether participants agree that it is complete and accurate) and qualitative (additional suggestions or comments). 7 Summary statistics (frequencies) were generated for participants demographics. Content analysis was used to analyze free-text responses: to remove out-of-scope suggestions, group similar suggestions together, and compare them to existing data points in the workflow. Data analysis and workflow updates were performed in duplicate by two independent analyzers (H.L.H. and P.G.).

### Ethics

This study is independent of national health services and does not require ethical approval interrogated via online Health Research Authority decision tool ( Supplementary Material B , available in the online version). 7 21

## Results

### Participants

The Delphi Round 1 was completed by a group of five consultant skull base surgeons. Two neurosurgeons at the National Hospital for Neurology and Neurosurgery, London, United Kingdom, and three ENT surgeons at the Royal National Throat, Nose and Ear Hospital, London, United Kingdom. Cumulatively, they had a median of 12.3 years and interquartile range (IQR) of 16.0 years of experience (IQR: 1 9.6 years; IQR: 25.5 years). The Delphi Round 1 was repeated four times during a 4-month period (October 2020–February 2021) until saturation.

The Delphi Round 2 was completed by nine neurosurgeons and eight ENT surgeons based at 10 centers across the United Kingdom. All contributing authors are specialist lateral skull base surgeons with an independent surgical practice in vestibular schwannoma surgery who are members of the BSBS (either neurosurgery or ENT). Cumulatively, they had a median of 13.9 years and IQR of 18.1 years of experience (IQR: 1 7.5 years; IQR: 3 25.5 years). Round 2 was repeated twice during a 3-month period (May–July 2021) until saturation. There was a 100% response rate and no attrition across both the Delphi Rounds.

### Translabyrinthine Approach

Five distinct phases were delineated as follows: (1) approach and exposure, (2) mastoidectomy, (3) internal auditory canal and dural opening, (4) tumor debulking and excision, and (5) closure. As with the retrosigmoid approach, the preoperative set-up and postoperative protocols were recognized as important, but not within the scope of this study.

#### Phase 1: Approach and Exposure

This phase consisted of four steps, beginning with the postaural curvilinear incision to expose the mastoid bone ( Table 2 ).

**Table 2:** Translabyrinthine operative workflow phase 1: approach and exposure

No.	Steps	Instruments	Technique error	Event error
1	Post aural curvilinear incision	Scalpel, monopolar, bipolar, periosteal elevator		
2	Creation of plane to posterior canal wall	Scalpel, monopolar, bipolar, periosteal elevator	•Incision of ear canal	•Cerebrospinal fluid (CSF) otorrhea
3	Hemostasis	Bipolar, monopolar, suction		
4	± Musculoperiosteal incision, elevation and retraction of flap	Scalpel, monopolar, bipolar, suture, bone wax, self-retaining retractor, periosteal elevator	•Inappropriately large or small or flap •Flap not retracted above root of zygomatic process •Incision of ear canal •Waxing emissary vein if very large and sigmoid sinus injury	•Hemorrhage •Inadequate exposure •CSF otorrhea

#### Phase 2: Mastoidectomy

This phase consisted of 13 steps, starting with an extended cortical mastoidectomy to give lateral petrous dissection, systematic three canal osseous labyrinthectomy, and completion of the labyrinthectomy ( Table 3 ).

**Table 3:** Translabyrinthine operative workflow phase 2: mastoidectomy

No.	Steps	Instruments	Technique error	Event error
1	Extended cortical mastoidectomy superiorly and posteriorly to give lateral petrous dissection	Drill and self-irrigating system (± cutting, ± diamond), bone wax	•Not enough access to IAC if mastoidectomy is limited •Dural sinus and emissary vein injury •Injury to middle fossa dura and temporal lobe •Injury to posterior canal wall	•Hemorrhage •Seizure •CSF otorrhea
2	Identification of sigmoid sinus (including retrosigmoid air cells) ± decompression if necessary	Drill and self-irrigating system (± cutting, ± diamond), bone wax	•Dural sinus and emissary vein injury •Injury to middle fossa dura and temporal lobe	•Hemorrhage •Air embolism •Sinus thrombosis •Seizure
3	Exenteration of all mastoid air cells	Drill and self-irrigating system (± cutting, ± diamond), bone wax		
4	Opening of antrum to view lateral semicircular canal and incus to project the position of the second genu and vertical parts of CN VII	Drill and self-irrigating system (± cutting, ± diamond), bone wax	•Failure to expose and locate these bony landmarks •CN VII injury •Injury to middle fossa dura and temporal lobe •Injury to posterior canal wall	•Hemorrhage •CN VII palsy •Seizure •CSF otorrhea
5	± Identification of digastric ridge			
6	Skeletonization of sigmoid sinus from sinodural angle ± bony island preserved on sigmoid sinus	Drill and self-irrigating system (± cutting, ± diamond), bone wax	•Insufficient skeletization of sigmoid sinus resulting in inability to compress sinus •Sigmoid sinus injury •Injury to middle fossa dura and temporal lobe	•Hemorrhage •Air embolism •Sinus thrombosis •Seizure
7	Skeletonization of middle fossa dura from zygomatic root to sinodural angel	Drill and self-irrigating system (± cutting, ± diamond), bone wax, Freers' elevator, Jansen–Middleton/Kerrison's rongeur, bipolar diathermy	•Coagulation injury •Superior petrosal sinus injury •Injury to middle fossa dura and temporal lobe	•Hemorrhage •Postoperative CSF leak •Seizure
8	Drilling of peri labyrinthine air cells to define three semicircular canals	Drill and self-irrigating system (± cutting, ± diamond), bone wax	•Sigmoid sinus injury •Injury to middle fossa dura and temporal lobe	•Hemorrhage •Seizure
9	Systematic three canal osseous labyrinthectomy	Drill and self-irrigating system (± cutting, ± diamond), bone wax	•Insufficient preservation of lateral and superior ampullated ends •CN VIII injury •Injury to middle fossa dura and temporal lobe	•Hemorrhage •CN VIII palsy •Seizure
10	Identification of the vestibule			
11	Drilling of Trautmann's triangle with identification of vestibular aqueduct and endolymphatic sac	Drill and self-irrigating system (± cutting, ± diamond), bone wax	•Vessel injury	•Hemorrhage
12	Widening of exposure of resulting in completion of labyrinthectomy	Drill and self-irrigating system (± cutting, ± diamond), bone wax	•Vessel injury	•Hemorrhage

Abbreviations: CN, cranial nerve; CSF, cerebrospinal fluid; IAC, internal auditory canal.

Note: We appreciate the exact order of the following steps will be surgeon and tumor characteristic dependent.

#### Phase 3: Internal Auditory Canal and Dural Opening

This phase consisted of 13 steps, from developing the inferior dissection by drilling out the retrofacial air cells to completion of dural dissection superiorly and inferiorly ( Table 4 ). The petrosal vein may be encountered superiorly. Consensus dictated that the petrosal vein may be coagulated and divided only if absolutely necessary to reduce the risk of venous infarct.

**Table 4:** Translabyrinthine operative workflow phase 3: internal auditory canal and dural opening

No.	Steps	Instruments	Technique error	Event error
1	Develop inferior dissection by drilling out retrofacial air cells	Drill and self-irrigating system (± cutting, ± diamond), bone wax	•Injury to CN VII •Injury to presigmoid posterior fossa dura •Injury to AICA	•Hemorrhage •CN VII palsy
2	Identification of jugular bulb ± skeletonization of jugular bulb if required	Drill and self-irrigating system (± cutting, ± diamond), bone wax	•Failure to adequately skeletonize the jugular bulb •Vascular injury if high riding bulb as needs to be delineated •CN VII injury •Injury to CN IX, X, XI if drill too deeply	•Insufficient access to internal acoustic meatus •Hemorrhage •CN VII, IX, X, XI palsy
3	Identification of cochlear aqueduct to decompress posterior fossa space if good CSF run-off			
4	Continuous drilling to reach the porous medially and thinning of tegmen	Drill and self-irrigating system (± cutting, ± diamond), bone wax	•Injury to middle or posterior fossa dura	
5	Defining cochlear aqueduct as inferior limit of dissection at that point	Drill and self-irrigating system (± cutting, ± diamond), bone wax	•Failure to identify and protect CN IX, X, XI in neural compartment of jugular foramen	•CN IX, X, XI palsy
6	Superior and inferior troughs to IAC drilled ± 200 to 270 degrees around the porous and laterally at the level of the fundus	Drill and self-irrigating system (± cutting, ± diamond), bone wax	•Failure to expose 270 degrees, failure to drill parallel to IAC •Dissect internal meatus gutter where CN VII commonly courses •CN VII injury •Air cell opening without repair •Injury to middle or posterior fossa dura	•CN VII palsy •CSF leak
7	Removal of bone with preservation of internal meatus dura	Microscope, bipolar, suction, microdissector, microscissors, Cottonoid patties, inside knife, sickle knife, 90 degree hook	•CN VII injury	•CN VII palsy
8	Reflection of superior and inferior vestibular nerves	Microscope, bipolar, suction, microdissector, microscissors, Cottonoid patties, inside knife, sickle knife, 90 degree hook	•CN VIII injury	•CN VIII palsy
9	Identification of CN VII at the fundus, superior to the transverse crest	Microscope, facial nerve stimulator	•Failure to identify CN VII	•CN VII palsy
10	Posterior fossa dural elevation, incision and opening, posteriorly first to facilitate CSF run off	Microscope, bipolar, suction, microdissector, microscissors, Cottonoid patties, inside knife, sickle knife, 90 degree hook	•Injury to cerebellum, sigmoid sinus, or petrosal vein •Injury to CN VII at the fundus	•Hemorrhage •Air embolism •Sinus thrombosis •CN VII palsy
11	Complete dural dissection superiorly and inferiorly	Microscope, bipolar, suction, microdissector, microscissors, Cottonoid patties, inside knife, sickle knife, 90 degree hook	•CN injury •Vessel injury	•Hemorrhage •CN palsy
12	Define interface of lateral part of tumor from cerebellum by dividing or dissecting the arachnoid mater	Microscope, bipolar, suction, microdissector, microscissors, Cottonoid patties, scalpel	•CN injury •Vessel injury	•Hemorrhage •CN palsy
13	Identification and protection of petrosal vein ± coagulation and division of petrosal vein only if absolutely necessary	Microscope, bipolar, suction, microdissector, microscissors, Cottonoid patties, scalpel	•Traction on petrosal vein •Injury to SCA •Sinus injury	•Venous infarct or hematoma •Air embolism •Sinus thrombosis

Abbreviations: CSF, cerebrospinal fluid; IAC, internal auditory canal; SCA, superior cerebellar artery.

#### Phase 4: Tumor Debulking and Excision

This phase consisted of 18 steps and begins with attempted identification of the facial nerve ( Table 5 ). Similar to the retrosigmoid approach, this phase describes the stepwise debulking of the tumor at the superior and inferior poles, with lateral–medial and medial–lateral dissection, and culminating in stepwise rolling and debulking of the tumor. Surgeon preference, intraoperative findings, and tumor characteristics define the exact order of the constituent steps within this phase. Further, depending on the patient's clinical history and presentation, a cochlear implant may be considered.

**Table 5:** Translabyrinthine operative workflow phase 4: tumor debulking and excision

	Steps	Instruments	Technique error	Event error
1	Posterior aspect of tumor stimulated for facial nerve	Microscope, facial nerve stimulator	•Failure to identify CN VII	•CN VII palsy
2	Posterior part of tumor coagulated and debulked	Microscope, facial nerve stimulator, bipolar, suction, microdissector, microscissors, Cottonoid patties, ultrasonic aspirator, tumor holding forceps, rongeur	•Inadequate hemostasis	•Hemorrhage
3	Tumor biopsy	Tumor holding forceps, rongeur		•Hemorrhage
4	Central core of tumor resected	Microscope, facial nerve stimulator, bipolar, suction, microdissector, microscissors, Cottonoid patties, ultrasonic aspirator, tumor holding forceps, rongeur	•Excessive traction on tumor •Injury to multiple cranial nerves, vessels, brain	•CN palsy •Hemorrhage
5	Inferior pole resection and separation from lower cranial nerves and vessels	Microscope, facial nerve stimulator, bipolar, suction, microdissector, microscissors, Cottonoid patties, ultrasonic aspirator, tumor holding forceps	•Injury to CN IX, X, XI •Injury to vessels: AICA, PICA •Incomplete tumor excision	•CN IX, X, XI palsy •Hemorrhage •Infarct •Labile heart rate and blood pressure intraoperatively
6	Identification of CN VIII at brainstem and dissection of arachnoid medially	Microscope, facial nerve stimulator, bipolar, suction, microdissector, microscissors, Cottonoid patties, scalpel	•Incorrect arachnoid plane •Perforating vessel injury •Injury to CN VII or VIII	•CN VII or VIII palsy •Brainstem, peduncle infarct
7	± Identification of dorsal cochlear nucleus for DNAP electrode if considering cochlear preservation	DNAP electrode		
8	± Cochlear implant in selected cases	Cochlear implant		
9	Identification of the root entry of CN VII which lies ventral and inferior to root entry of CN VIII	Microscope, facial nerve stimulator, bipolar, suction, microdissector, microscissors, Cottonoid patties, scalpel	•Vessel injury •Injury to CN VII	•Hemorrhage or infarct •CN VII palsy
10	± FREMAP electrode	FREMAP electrode		
8	Lateral tumor resection	Microscope, facial nerve stimulator, bipolar, suction, microdissector, microscissors, Cottonoid patties, ultrasonic aspirator, tumor holding forceps	•Failure to identify ascending CN VII •Injury to superior cerebellar artery, anterior inferior cerebellar artery •Incomplete tumor excision	•CN VII palsy •Hemorrhage
9	Superior pole resection	Microscope, facial nerve stimulator, bipolar, suction, microdissector, microscissors, Cottonoid patties, ultrasonic aspirator, tumor holding forceps	•Injury to CN V or VII •Injury to petrosal vein or SCA •Incomplete tumor excision	•CN V, VII palsy •Hemorrhage •SCA infarct
10	Dissection of tumor capsule from CN V	Microscope, facial nerve stimulator, bipolar, suction, microdissector, microscissors, Cottonoid patties, scalpel	•Injury to CN IV or V •Injury to SCA	•CN IV or V palsy •SCA infarct
11	Locate fundus of IAM and dissect superior vestibular nerve as laterally as possible	Microscope, facial nerve stimulator, bipolar, suction, microdissector, microscissors, Cottonoid patties, scalpel	•Injury to CN VII •Incomplete tumor excision	•CN VII palsy
12	Ensure preservation of cochlear nerve and the dura and capsule of anterior part of IAM to allow lateral to medial dissection	Microscope, facial nerve stimulator, bipolar, suction, microdissector, microscissors, Cottonoid patties, scalpel	•Insufficient rotation of operating table to best visualize dissection plane •CN VII injury	•CN VII palsy
13	Continue dissection with lateral to medial dissection to the porous	Microscope, facial nerve stimulator, bipolar, suction, microdissector, microscissors, Cottonoid patties, scalpel	•Failure to keep CN VII visualized at all times •CN VII injury •Failure to maintain plane between tumor and CN VII •Incomplete tumor excision	•CN VII palsy
14	Resection of tumor in the CPA until lateral–medial and medial–lateral dissections to join together	Microscope, facial nerve stimulator, bipolar, suction, microdissector, microscissors, Cottonoid patties, ultrasonic aspirator, tumor holding forceps	•CN injury •Vessel injury •Incomplete tumor excision	•Hemorrhage •CN palsy
15	± Division of CN VIII and continuation of intracapsular component to minimize damage to CN VII	Microscope, facial nerve stimulator, bipolar, suction, microdissector, microscissors, Cottonoid patties, scalpel		
16	Aim for total or near total tumor excision pending tumor size/preoperative planning to preserve CN VII at most vulnerable (at porous)	Microscope, facial nerve stimulator, bipolar, suction, microdissector, microscissors, Cottonoid patties, scalpel		
17	Hemostasis	Bipolar, fibrin sealant, oxidized cellulose matrix, Cottonoid patties	•Incomplete hemostasis	•Hematoma
18	± In circumstance when facial nerve is not preserved, perform facial nerve graft (proximal and distal stump anastomosis using nerve ± sural or greater auricular nerve)	Scalpel, monopolar, retractor, microscope, suture	•Incomplete anastomosis	•CN VII palsy

Abbreviations: AICA, anterior inferior cerebellar artery; CN, cranial nerve; CPA, cerebellopontine angle; DNAP, dorsal cochlear nucleus action potential; FREMAP, Facial nerve root exit zone–elicited compound muscle action potential; IAM, internal auditory meatus; IQR, interquartile range; PICA, posterior inferior cerebellar artery; SCA, superior cerebellar artery.

Note: We appreciate the exact order of the following steps will be surgeon and tumor characteristic dependent.

#### Phase 5: Closure

This phase consisted of 11 steps ( Table 6 ), encompassing hemostasis, packing of the Eustachian tube and middle ear, and multilayer closure of the wound. There was variability in the substance to pack the Eustachian tube with (bone wax, muscle, periosteum, and dural substitute) and the location for harvesting a tissue graft (abdomen, leg, and fascia lata).

**Table 6:** Translabyrinthine operative workflow phase 5: closure

No.	Steps	Instruments	Technique error	Event error
1	CN VII stimulation to confirm response at low level (0.05 mA)	Facial nerve stimulator	•No stimulation	•CN VII palsy
2	Hemostasis	Bipolar, fibrin sealant, oxidized cellulose matrix, Cottonoid patties	•Incomplete hemostasis	•Hematoma
3	Removal of incus. Tensor tympani muscle divided to allow malleus to be reflected laterally. Visualize Eustachian tube opening in middle cleft	Bone wax, hook, crocodile forceps, Hughes elevator, microscissors	•Failure to protect CN VII •Dislocation of stapes	•CN VII palsy •CSF leak
4	Pack Eustachian tube and middle ear	Bone wax, ± muscle, periosteum, or dural substitute + tissue sealant, to middle ear and aditus	•Failure to protect CN VII •Dislocation of stapes •Tympanic membrane perforation	•CN VII palsy •CSF leak
5	± Wax off the vestibule and air cells around facial nerve	Bone wax		•CSF leak
6	Harvesting of graft (± fat from abdomen or leg, ± fascia lata)	Scalpel, monopolar, bipolar, forceps, scissors		
7	Tissue graft inserted into petrosal cavity	Forceps, bipolar, graft (± fat or fascia lata), ± suture dura over Trautman triangle	•Fat packing into CPA or aggressive packing •Loose packing of fat graft	CN IX, X, XI palsy •Hemorrhage •CSF leak
8	Temporalis closure	Suture, ± dural sealant glue	•Incomplete closure	•CSF leak •Pseudomeningocoele
9	Removal of skin traction with silk stiches			
10	Skin closure	Suture, clips	•Poor opposition of skin edges	•Wound infection • CSF leak
11	± Abdominal wall closure in layers ± placement of suction drain	Suture, clips	•Poor opposition of skin edges	•Wound infection

Abbreviations: CN, cranial nerve; CPA, cerebellopontine angle; CSF, cerebrospinal fluid.

## Discussion

### Principal Findings

We present Part 2 of a series that generated a consensus-derived codified operative workflow for the translabyrinthine approach to vestibular schwannoma. Each workflow considers the phases, steps, technique errors, and event errors of the operation. The operative workflow was achieved through national collaboration with the BSBS following an open invitation to all members to participate. This comprised 17 independently practicing neurosurgeons and ENT surgeons from 11 centers across the United Kingdom.

The translabyrinthine approach operative workflow comprises the following five distinct phases with a total of 59 individual steps: (1) approach and exposure, (2) mastoidectomy, (3) internal auditory canal and dural opening, (4) tumor debulking and excision, and (5) closure. The translabyrinthine approach contains two more phases and 19 more steps than the retrosigmoid approach. 1

The codified operative workflow for the translabyrinthine approach provides an illustrative example of how surgical procedures can be deconstructed. The presented workflow is foundational research for future work exploring the application of artificial intelligence to surgery or comparative surgical research which may unlock a new phase in surgical training and technical improvement.

### Operative Workflows to Facilitate Comparative Surgical Research

There is little high-quality evidence comparing both surgical and nonsurgical factors at reducing morbidity in vestibular schwannoma surgery. Bartek et al 22 presented a national, short-term (30 days) surgical outcome registry, focusing on tumor size and patient age. This example does not consider granular technical nuance, such as the use of bone cement or bone wax when sealing the mastoid air cells. Selleck et al 23 presented a single-center retrospective cohort evaluating the use of mesh cranioplasty versus periosteal closure at mitigating cerebrospinal fluid leaks. Although a granular question, it is low-quality evidence and the findings have not been generalized. Therefore, a systematic operative workflow can provide the framework to ask which specific techniques may result in improved outcomes.

The morbidity and mortality associated with vestibular schwannoma resection has decreased in modern practice from the early pioneers. 24 25 26 Despite advances in practice and improving mortality rates, the morbidity remains high for these common tumors 27 which can significantly impair a patient's quality of life. For a complex procedure, practiced by experienced surgeons, any small incremental improvement in technique may result in improved outcomes. As such, the workflows in the present study provide an objective consensus in the current variability within practice and a foundation in which to develop further research questions. For example, for each variation in technique outlined within our workflows, we could further explore how many surgeons perform which technique and correlate this with outcomes. This could drive a national or international audit process to provide guidance on how the operation should be performed in the future. The operative workflow could also assist in the generation of performance metrics for each procedure.

### Computer Vision and Operative Workflows

The parcellation of operative videos can be achieved through computer vision, an artificial intelligence-driven algorithm that automatically detects the phase and step of an operation. 28 The principal limitation to workflow analysis is the labor-intensive labeling and segmentation of operations into constituent phases, steps, and errors; however, this process can be automated (or semiautomated), using machine learning techniques. 28 29 30 The effectiveness of such automation is dependent on the generation of a codified, comprehensive operative workflow to train deep neural networks to recognize the phases, steps, instruments, and errors of an operation. 7 Our group has previously demonstrated that a machine learning algorithm can accurately and autonomously identify the various phases and steps of an endoscopic transsphenoidal resection of pituitary adenomas. 7 If a machine learning algorithm can identify the correct phase and step of a vestibular schwannoma resection and compare multiple operative videos against outcomes, it might identify subtleties within technique that could improve functional outcomes or reduce surgical complications. It might also permit the ability to separate between essential and nonessential steps or highlight specific steps that are with high risk during an operation. It is unclear presently if machine learning will be able to identify the phases and steps of a vestibular schwannoma resection accurately and autonomously, due to heterogeneity between technique and order of phases. We plan to use this workflow to test this hypothesis in future work.

## Strengths and Limitations

This is the first expert, consensus-derived operative workflow for the translabyrinthine approach. Our methodology follows precedence within in the literature. Further, both operative workflows are presented with concordant nomenclature, and share homogenous descriptions of the steps, instruments, and errors if appropriate. This will allow greater transparency and comparison between approaches, and indeed further scope to develop the workflows in the future. However, our methodology did not deconstruct which phase and steps were performed by neurosurgeons and ENT surgeons, nor did we include the use of endoscopy, for example, endoscopic exploration of the internal acoustic canal to identify potential mastoid cells opening before closure. This is likely different in each center based on local expertise. This will require consideration when trying to evaluate outcomes in future work.

## Conclusion

We present Part 2 of a national, multicenter, consensus-derived codified operative workflow for the translabyrinthine approach to vestibular schwannomas. The five phases contain the operative steps, instruments, technique errors, and event errors. The codified translabyrinthine approach presented in this manuscript can serve as foundational research for future work, such as the application of artificial intelligence to vestibular schwannoma resection and comparative surgical research.
